# Editorially

**Published:** 1887-12

**Authors:** 


### EDITORIALLY.

Owing to the great amount of other matter which demands a place, the usual leading editorial for this number is omitted. We are well aware that the average reader likes to see something in each number from the editor, even if it be not very brilliant. His opinions may be crude, his judgment feeble and his reasons vapid, but the editorial sauce seasons them, and they are accepted in place of better reading. As plain W. C. B., what we may say is of little moment; but as the editor of the INDEPENDENT PRACTITIONER, it may be read and tolerated, and perhaps we have at times trespassed too freely upon this indulgence. But like pulpit orators, we have our hearers where they cannot talk back, and the privilege is too dear to be lightly relinquished. At any rate, we are in no mood to promise amendment for the future.

